# Genome Sequence of Foot-and-Mouth Disease Virus of Serotype O Lineage Ind-2001d Isolated from Cattle in Mongolia in 2015

**DOI:** 10.1128/genomeA.01244-17

**Published:** 2017-11-09

**Authors:** Tatsuya Nishi, Gerelmaa Ulziibat, Buyantogtokh Khanui, Odonchimeg Myagmarsuren, Kazuki Morioka, Makoto Yamakawa, Katsuhiko Fukai

**Affiliations:** a Exotic Disease Research Unit, Division of Transboundary Animal Diseases, National Institute of Animal Health, National Agriculture and Food Research Organization, Josui-honcho, Kodaira, Tokyo, Japan; b State Central Veterinary Laboratory, Khan-Uul District, Ulaanbaatar, Mongolia

## Abstract

We report the whole-genome sequence of the foot-and-mouth disease virus (FMDV) O/MOG/BU/2-7/2015 isolated in Mongolia in 2015. This virus is closely related to isolates identified in Southeast Asia in 2015 and is classified under the O/ME-SA/Ind-2001d lineage. This is the first detection of an FMDV of this lineage in Mongolia.

## GENOME ANNOUNCEMENT

Foot-and-mouth disease virus (FMDV) is classified under the *Aphthovirus* genus within the *Picornaviridae* family. Its genome is composed of a single strand of positive-sense RNA of approximately 8.4 kb in length, which is divided into S and L fragments by a poly(C) sequence at the 5′ untranslated region (UTR). FMDV is categorized into seven immunologically distinct serotypes (O, A, C, Asia 1, and Southern African Territories 1 to 3), each of which can be divided into a variety of genetically distinct topotypes and lineages (1).

The FMDV serotype O topotype Middle East-South Asia lineage Ind-2001 (O/ME-SA/Ind-2001) was detected and first defined in 2001 and has been the dominant lineage in the Indian subcontinent since 2008 (2, 3). FMDV of the O/ME-SA/Ind-2001d sublineage has been confirmed in countries in the Middle East, West Eurasia, and North Africa since 2013 and was detected in Laos, Vietnam, Myanmar, Thailand, Russia, China, and the Republic of Korea from 2015 to 2017 (4–6).

In the present study, epithelial samples were collected from cattle that showed clinical symptoms of FMDV in Bayan-Ulgii province in Mongolia in March 2015. The FMDV O/MOG/BU/2-7/2015 was isolated from samples according to the World Organisation for Animal Health (OIE) manual (7) and plaque cloned using ZZR-127 cells (8). Viral RNA was extracted and the L-fragment gene, of approximately 7.7 kb, was amplified by PCR using four pairs of FMDV-specific primers. The nucleotide sequences were analyzed using the Ion PGM system (Life Technologies, Inc., Carlsbad, CA, USA) as previously described (9).

The L-fragment sequence of O/MOG/BU/2-7/2015 was 7,754 nucleotides (nt) in length, and included a 5′ UTR of 685 nt, a single open reading frame (ORF) of 6,999 nt, and a 3′ UTR of 70 nt. The ORF was predicted to encode a polyprotein of 2,333 amino acids composed of four structural proteins, VP1 to VP4, and eight nonstructural proteins, L, 2A to 2C, and 3A to 3D. This virus isolate is the most closely related to O/BAN/GO/Ka-236(Pig)/2015 (GenBank accession no. KX712091) in the public database, with a 98.6% nt identity rate and no indels.

In conclusion, the FMDV O/ME-SA/Ind-2001d was isolated from epithelial samples following an outbreak in 2015 in the western part of Mongolia. Our findings confirm the first incursion of FMDV O/ME-SA/Ind-2001d into East Asia. FMDV O/ME-SA/Ind-2001d has continued to spread into the Eurasian and African continents, indicating the need for epidemiological information and monitoring of this lineage to develop appropriate control strategies against this disease.

### Accession number(s).

The genome nucleotide sequence of O/MOG/BU/2-7/2015 has been deposited in GenBank under accession no. LC320038.
